# Gate Metal Defect Screening at Wafer-Level for Improvement of HTGB in Power GaN HEMT

**DOI:** 10.3390/mi16111260

**Published:** 2025-11-06

**Authors:** Yu-Ting Chuang, Niall Tumilty

**Affiliations:** 1 MossFloat Ltd., Hsinchu City 30053, Taiwan; 2 International College of Semiconductor Technology, National Yang Ming Chiao Tung University, Hsinchu City 30010, Taiwan; ntumilty@nycu.edu.tw

**Keywords:** p-GaN HEMTs, gate metal defect, HTGB

## Abstract

The increasing market demand for high-power and high-frequency applications necessitates the development of highly reliable Gallium Nitride (GaN) High-Electron-Mobility Transistors (HEMTs). While GaN offers superior performance and efficiency over traditional silicon, gate-related defects pose a significant reliability challenge, often leading to premature device failure under stress. Traditional High-Temperature Gate Bias (HTGB) testing is effective but time-consuming and costly, particularly when defects are only identified post-packaging. This study focuses on developing an effective wafer-level screening methodology to mitigate the financial burden and reputational risk associated with late-stage defect discovery. Failure analysis of an HTGB premature failure revealed a gate metal deposition defect characterized by identical elemental composition to the bulk metal, suggesting a small-volume structural anomaly. Crucially, a comparative analysis showed that Forward Gate Current (I_GON_) is an insensitive screening metric due to high inherent gate leakage through the passivation layer. In contrast, the Reverse Gate Current (I_GOFF_) exhibited sensitivity, particularly under the tensile stress induced by package molding, which is attributed to the piezoelectric effect altering the depletion region width beneath the p-GaN gate. Based on this observation, a multi-pulse I_DSS_ test was developed as a wafer-level screen. This method successfully amplified the subtle electrical field perturbations caused by the gate defect. After screening 231 dies using the new methodology, zero failures were recorded after 1000 h of HTGB stress, a significant improvement over the initial failure rate of 0.43% (1 out of 231). This work demonstrates that early, sensitive wafer-level screening of gate defects is indispensable for optimizing manufacturing yield and enhancing long-term device reliability.

## 1. Introduction

The increasing demand for high-power and high-frequency electronic devices has driven the development of Gallium Nitride (GaN) High-Electron-Mobility Transistors (HEMTs). GaN HEMTs have emerged as a promising technology for next-generation power electronics applications, offering superior performance, efficiency, and reliability compared to traditional silicon-based devices [1,2]. However, as GaN HEMTs are increasingly used in high-power and high-frequency applications, the importance of ensuring their reliability and robustness cannot be overstated.

Reliability is a critical aspect of GaN HEMT performance, as it directly impacts the overall system reliability and maintenance costs [3]. One of the most critical reliability concerns for GaN HEMTs are the gate-related defects, which can lead to device failure and reduced lifespan [4]. High-Temperature Gate Bias (HTGB) testing has become a widely accepted method for evaluating the reliability of GaN HEMTs under extreme operating conditions. HTGB testing involves applying a high gate bias voltage to the device at elevated temperatures, simulating the harsh conditions encountered in real-world applications [5,6].

The importance of HTGB testing lies in its ability to accelerate the degradation mechanisms that occur in GaN HEMTs, allowing for the identification of potential failure modes and the evaluation of device reliability [7,8]. However, HTGB testing can be time-consuming and costly, making it challenging to implement in high-volume manufacturing environments. Moreover, the risk of field returns due to gate-related defects can be significant, resulting in substantial costs and reputational damage for manufacturers.

**Figure 1:**
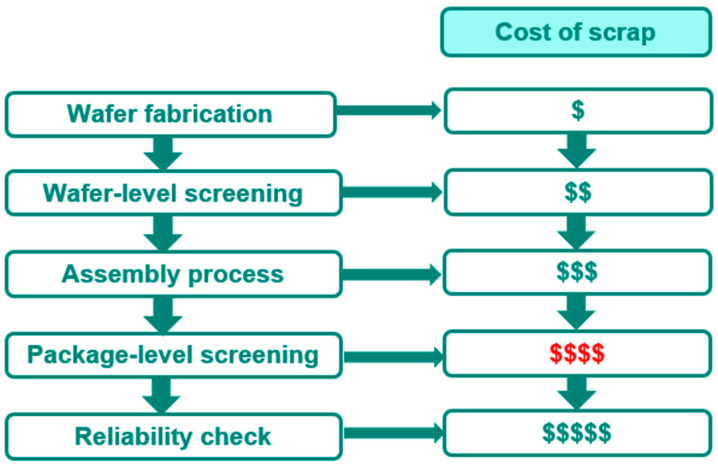
Manufacture flow with cost of scrap illustrated. The cost of manufacturing covers wafer fabrication, wafer-level screening, assembly process, package-level screening, and reliability check. The cost of scrap shall decrease as early as the defect can be detected.

Wafer-level screening of gate-related defects offers a promising solution to this challenge. By identifying and eliminating defective devices at the wafer-level, manufacturers can save significant costs associated with packaging, testing, and field returns as Figure 1 shown. Wafer-level screening can also reduce the risk of field returns, ensuring that only reliable devices are shipped to customers. This approach can also enable the development of more robust and reliable GaN HEMTs, as manufacturers can focus on optimizing device design and manufacturing processes to minimize gate-related defects. This work focuses on investigating the importance of reliability in power GaN HEMTs, with a focus on wafer-level screening of gate-related defects.

## 2. Materials and Methods

**Figure 2:**
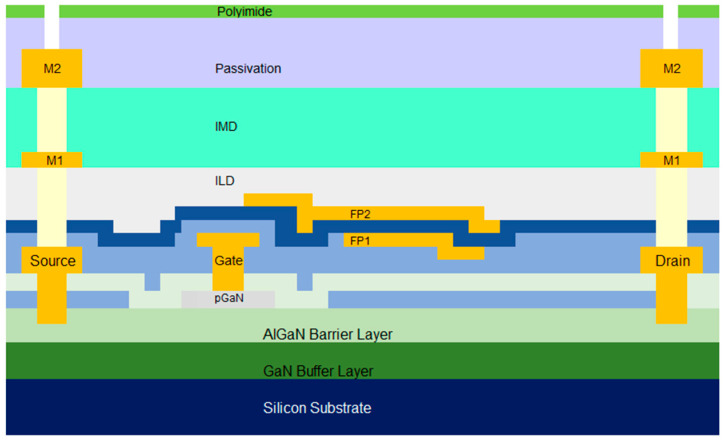
A cartoon structure of a typical power GaN HEMT.

**Figure 3:**
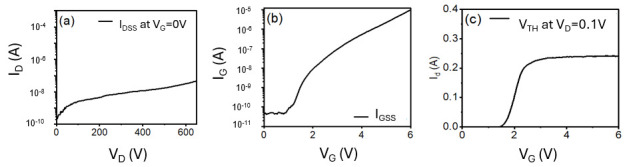
Typical (**a**) I_D_-V_D_ at V_D_ = 0~650 V, (**b**) I_G_-V_G_ at V_G_ = 0~6 V, and (**c**) I_D_-V_G_ at V_D_ = 0.1 V curves.

Figure 2 illustrates the cartoon cross-section of commercially available p-GaN power HEMTs. The epitaxial structure consists of a super-lattice GaN buffer layer on top of the silicon substrate. An AlGaN barrier layer stacks on top of the super-lattice GaN buffer followed by a p-GaN layer. Figure 3 shows the typical I–V curve of the GaN HEMTs.

**Figure 4:**
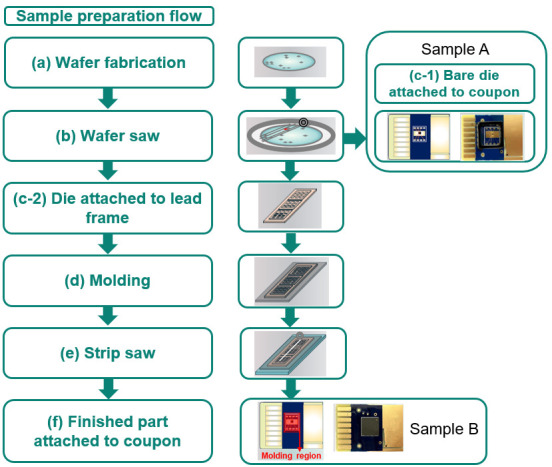
The manufacturing flow from wafer processing to final sample preparation. (**a**) The process begins with device fabrication in the foundry. (**b**) The fabricated wafers are transferred to the assembly house for wafer dicing. Two distinct packaging routes are then implemented. (**c-1**) Sample A (Non-Molded Assembly): Bare dies are picked and attached directly to the coupons. (**c-2**) Sample B (Molded Package Assembly): Dies are picked and attached to a lead frame. (**d**) The assembly is then subjected to the molding process, where the capsule is filled with molding compound. (**e**) The packaged parts are separated from the strips. (**f**) Packaged parts are mounted to the coupon for characterization and reliability tests.

Figure 4 illustrates the major manufacturing steps from wafer fabrication to the final packaged parts. The process commences with wafer manufacturing at the foundry, as depicted in Figure 4a. The fabricated wafers are subsequently transferred to the assembly house for wafer sawing (Figure 4b).

Sample A: Non-Molded Assembly

For Sample A, devices are picked from the dicing tape following wafer sawing and are directly attached to coupons (Figure 4(c-1)). A visual representation of Sample A is provided adjacent to Figure 4(c-1).

Sample B: Molded Package Assembly

The production of Sample B incorporates molding processes. Bare dies are first picked and attached to a lead frame using die attach material, as shown in Figure 4(c-2). This is followed by the molding process (Figure 4d), which involves filling the mold cavity with a molding compound and curing it in an oven. The packaged parts are then cleaved from the strips using a strip saw technique (Figure 4e) and subsequently attached to coupons (Figure 4f). An image of the resulting Sample B is presented above Figure 4f.

**Figure 5:**
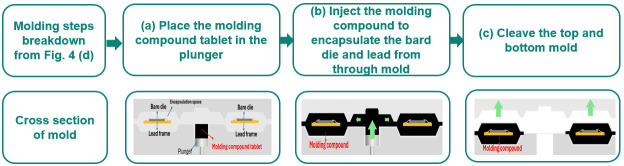
The molding procedure, detailed in Figure 4d, comprises three key steps for robust encapsulation. (**a**) The molding compound tablet is accurately placed into the plunger cavity. (**b**) The molten molding compound is injected into the mold, completely encapsulating the entire volume containing the bare die and lead frame. (**c**) The top portion of the mold is lifted, allowing the packaged parts to be cleaved for final processing.

The specific steps of the molding process detailed in Figure 4d are further elaborated in Figure 5b and can be delineated into three primary stages. The molding compound tablet is loaded into the plunger as illustrated in Figure 5a. The molding compound is injected into the capsule, fully encapsulating the bare die, bonding wires, and lead frame as shown in Figure 5b. The molding goes through a curing process to solidify the molding, which introduces external mechanical stress. The upper mold lifts off, and the encapsulated component is ready for cleaving as shown in Figure 5c.

## 3. Results and Discussion

**Figure 6:**
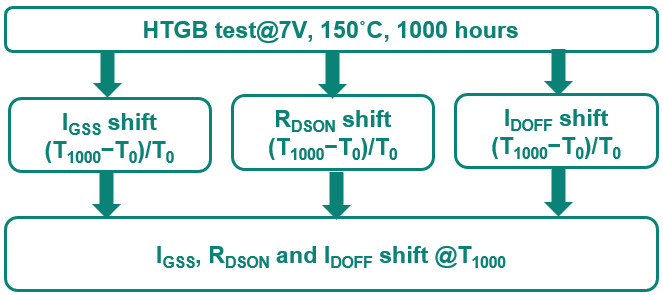
HTGB test comparison for typical power GaN HEMT.

**Figure 7:**
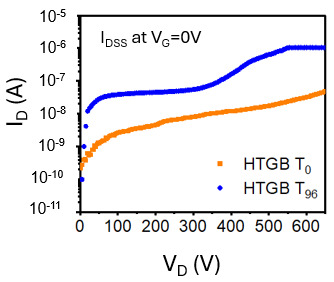
I_D_-V_D_ at V_G_ = 0 V characteristics for an HTGB fail part.

To assess the reliability of the device, a High-Temperature Gate Bias (HTGB) test was conducted under controlled conditions of V_G_ = 7 V, 150 °C, and 1000 h, as depicted in Figure 6. The key performance indicators for this test are the shifts in I_GSS_ (Gate Source Leakage Current), R_DSON_ (ON Resistance), and I_DSS_ (Drain Source Leakage Current). As a result, 1 part fails out of 231 testing parts after 96 h of stress. Figure 7 illustrates the I_D_-V_D_ curve after 96 h of HTGB stress, revealing an increase in drain leakage current, which indicates device failure.

**Figure 8:**
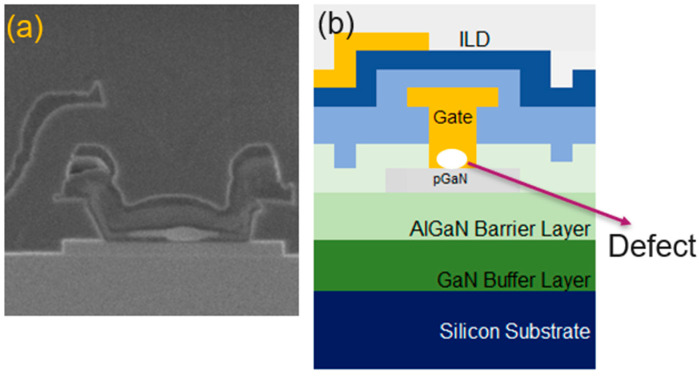
FIB hot spot. (**a**) Cross-section (**b**) Cartoon illustration of corresponding defect at bottom of gate metal.

To investigate the cause of failure, decapsulation hot spot analysis is performed. The top metal layer was removed to enhance the likelihood of detecting a hot spot. By sweeping I_DOFF_ to V_D_ = 650 V, an InGaAs hot spot was identified, and a cross-sectional analysis was conducted at the hot spot location, as shown in Figure 8a, to further elucidate the failure mechanism.

The Focused Ion Beam (FIB) cross-section in Figure 8a reveals a defect at the bottom of the gate metal, which is identified as a hot spot. A simplified illustration in Figure 8b helps to clarify the finding, indicating that the defect is located within the gate metal and is attributed to the gate metal deposition process. A detailed material characterization of the observed gate metal defect was performed using Scanning Electron Microscopy with Energy-Dispersive X-ray Spectroscopy (SEM-EDS). The analysis, conducted at an accelerating voltage of 5 keV, confirmed that the elemental composition of the defect site was spectroscopically identical to the surrounding bulk gate metal. This result strongly suggests that the defect volume is below the characteristic X-ray generation volume for the smallest beam energy, which is 5 keV. Specifically, the X-ray interaction volume at 5 keV is significantly larger than the defect itself, causing the resultant spectrum to be dominated by the underlying or surrounding bulk material.

The gate electrode itself features a multi-layer, or “sandwich,” composition, consisting of three distinct metal layers, as Figure 8a shows. These layers are sequentially deposited within a single, high-vacuum sputtering tool, utilizing separate, dedicated process chambers to ensure purity and control over individual layer properties. Given this integrated, high-vacuum manufacturing flow, introducing an in situ optical scanning or inspection step between the deposition of each metal layer is highly impractical and technically inadvisable. Any such interruption to the vacuum integrity, even for a brief period, would inevitably expose the newly deposited metal surface to ambient conditions, leading to rapid oxidation. This interfacial oxidation, in turn, severely compromises the crucial inter-layer adhesion, potentially creating new, more severe reliability issues.

To mitigate the current defectivity while maintaining process integrity, we have already enhanced the deployment of off-line monitoring and statistical process control (SPC) techniques during the metal deposition sequence. This allows for a more sensitive and rapid detection of incipient defects. However, despite these improvements in process control monitoring, a comprehensive review of the reliability data indicates that more stringent, testing methodologies and preventive measures are critically required across the entire fabrication module. These additional mechanisms are necessary to further suppress the underlying defect generation rate and achieve a substantially reduced, industry-leading long-term device failure rate.

**Figure 9:**
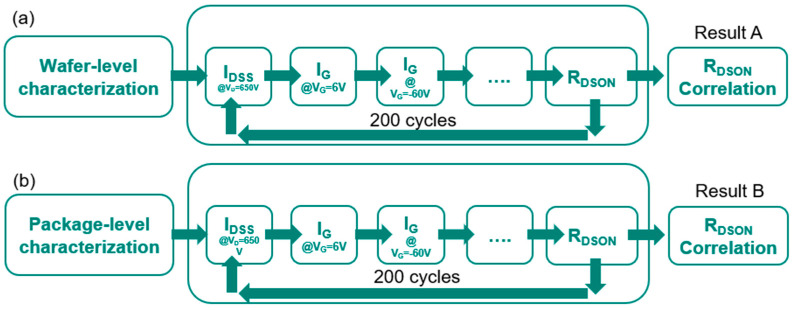
Schematics of standard R_DSON_ monitor loop-run characterizations to analyze the (**a**) on-wafer characteristics and (**b**) packaged device characteristics.

**Figure 10:**
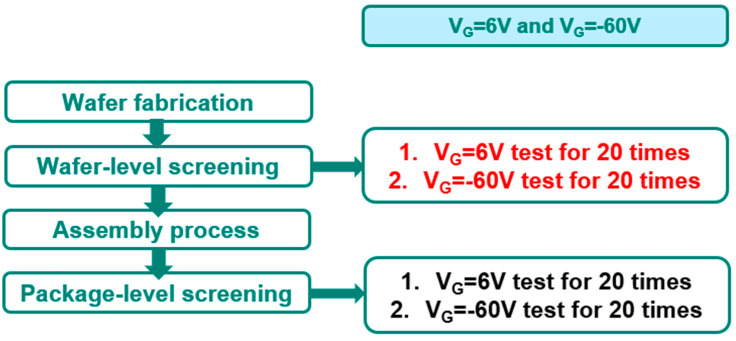
V_G_ = 6 V and V_G_ = −60 V pulse tests are performed 20 times in engineering samples in both wafer-level and package-level, then they are traced back for the correlation study of gate metal defect screening.

Testing data correlation is deployed. The looping test is our standard wafer-level reliability test for the R_DSON_ monitor, as shown in Figure 9. The major items in the loop-run test are I_DSS_ at V_D_ = 650 V, V_G_ at V_G_ = 6 V, V_G_@V_G_ = −60 V and R_DSON_. The loop-run testing items go through 200 cycles and ((R_DSON,T=200_ − R_DSON,T=0_)/R_DSON,T=0_) is calculated for the R_DSON_ monitor as result A for wafer-level characterization (Figure 9a). The same loop-run testing items go through 200 cycles and ((R_DSON,T=200_ − R_DSON,T=0_)/R_DSON,T=0_) is calculated for the R_DSON_ monitor as result B for package-level characterization (Figure 9b). To screen out the gate metal defect, a loop-run testing data correlation approach is employed and associated with the gate-related testing items, such as V_G_ = 6 V and V_G_ = −60 V. As a result, the testing data reveal a correlation with the reverse gate bias test at V_G_ = −60 V. A series of 20 sequential tests are conducted at both wafer level (Sample A) and package-level (Sample B), with a focus on the changes in I_G_ at V_G_ = −60 V between the 1st and 3rd tests, as well as between the 18th and 20th tests, as shown in Figure 10.

**Figure 11:**
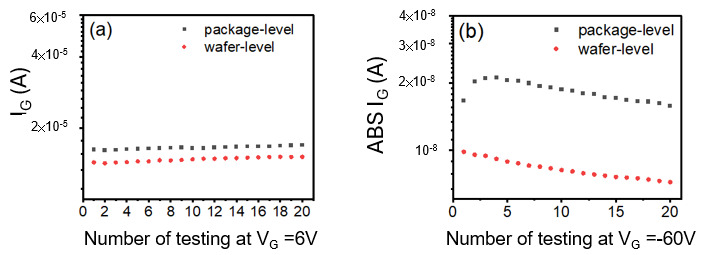
Comparison of T96 fail part testing data from Figure 10. (**a**) I_GON_ at V_G_ = 6 V. (**b**) I_GOFF_ at V_G_ = −60 V.

A lack of correlation was observed between the Forward Gate Current (I_GON_), measured at a gate voltage (V_G_) of 6 V, and the number of subsequent pulse test cycles, for both the wafer-level (Sample A) and package-level (Sample B) specimens, as illustrated in Figure 11a. This insensitivity is primarily attributed to the inherent high gate leakage current characteristic of these devices. This leakage stems from the relatively small energy barriers present at two critical interfaces: the Two-Dimensional Electron Gas (2DEG)/Aluminum Gallium Nitride (AlGaN) interface and the AlGaN/passivation layer interface. Consequently, the measured Forward Gate Current does not predominantly reflect the electrical state of the gate metal itself, but rather the total leakage current flowing through the passivation and overlying dielectric layers. Therefore, under forward gate bias conditions, the measured I_GON_ is not a sufficiently sensitive indicator for detecting subtle gate metal defects at either the wafer or package-level, rendering it ineffective as a screening metric, as confirmed by the data presented in Figure 11a.

In contrast, when the gate bias is reversed, electrons are injected from the gate metal into the passivation. The presence of a substantial energy barrier, particularly under tensile stress, leads to two possible mechanisms for electron injection: Fowler–Nordheim tunneling and thermionic emission. Once injected, the electrons follow specific leakage paths through the passivation, AlGaN, and GaN before being collected at the ohmic contact [9,10]. The results presented in Figure 11b reveal a significant difference in the delta values between the package-level (Sample B) and wafer-level (Sample A) tests for the HTRB T_96_ fail part taken before the HTGB stress. Initially, the I_G_ values become more negative for the first three tests at the package level (Sample B). However, after the third pulsed test, the changes in I_G_ versus the number of testing times become consistent with the accumulation of traps in AlGaN, GaN and passivation. The increase in traps is correlated with the number of testing times or stress; therefore, once the control of the gate with a gate metal defect drifts, it can be detected with a subtle change in trap accumulation with reverse gate bias under tensile package-level stress.

**Figure 12:**
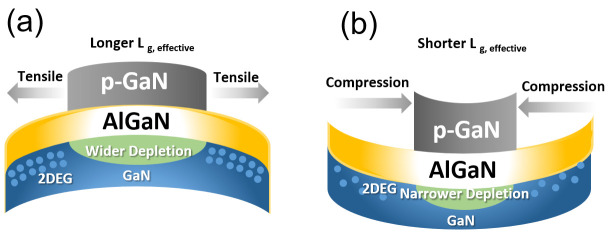
Cartoon illustration of piezoelectric effect on GaN depletion under pGaN. (**a**) AlGaN under Tensile Strain: tensile stress widens the depletion region and lengthens the effective channel length. (**b**) AlGaN under Compressive Strain: compressive stress narrows the depletion region and shortens the effective channel length.

This disparity can be explained by the piezoelectric effect on GaN High-Electron-Mobility Transistors (HEMTs), as illustrated in Figure 12a,b. In the context of AlGaN, compressive stress is expected to occur due to lattice and thermal mismatch after epitaxy growth using MOCVD [11]. However, the mechanical stress induced by the molding compound can transform the compressive stress into tensile stress in AlGaN [12]. This transformation leads to a longer effective channel length and a wider depletion region in devices with AlGaN under tensile strain, resulting in a lower I_G_ at reverse stress [13,14,15], as Figure 12a shows.

**Figure 13:**
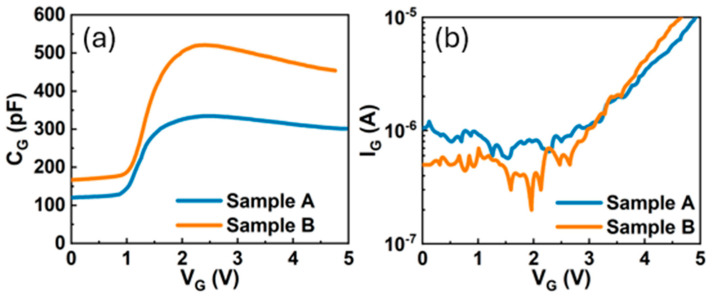
Typical (**a**) C_G_-V_G_ and (**b**) I_G_-V_G_ characteristics in Sample A and Sample B.

As shown in Figure 13a,b, Sample B exhibits a larger gate capacitance (C_G_) at low gate voltage (V_G_ < 1 V) and a lower gate leakage current (I_G_) for V_G_ < 2 V when compared to Sample A. These observations suggest that Sample B possesses a wider depletion region beneath the p-GaN gate, resulting in a reduced gate leakage current in the off-state (I_GOFF_) below 2V. This characteristic is hypothesized to correlate with variations in the width of the depletion region under the p-GaN gate, which is induced by mechanical stress originating from the packaging process.

**Figure 14:**
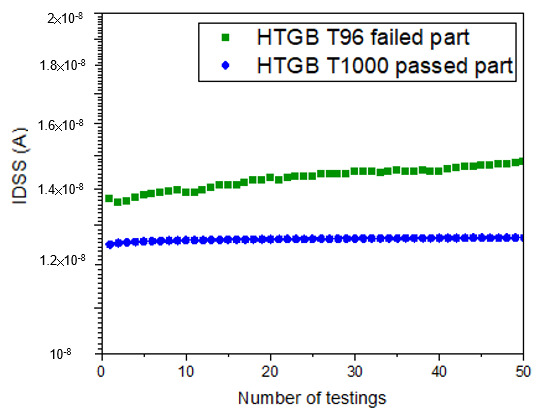
I_DSS_ test at V_D_ = 650 V. Repeat the testing condition for 50 times and plot the number of tests vs. I_DSS_ for HTGB T96 failed part and HTGB T1000 passing part.

Allowing the die with a gate metal defect to proceed in the manufacturing supply chain increases the cost of scrap at a later stage, as Figure 1 shows; therefore, a wafer-level screening methodology is needed. At the wafer-evel (Sample A), the depletion region under the p-GaN gate is narrower, as Figure 12b shows, making it more challenging to detect changes in gate controllability, such as the discrepancy shown in Figure 12. Since there is a subtle change in the leakage current at V_G_ = −60 V at the package-level, as Figure 12a shows, with high electrical field at the gate edge, the leakage current may also have a subtle change that can be detected by repeating the measurement of I_DSS_ with high electrical field at the gate edge. After going through several types of testing sequencing, repeating I_DSS_ at V_D_ = 650 V is the most promising screening test plan with 50 times at the wafer-level. The number of tests is plotted on the x-axis and that of I_DSS_ readings on the y-axis and we see that I_DSS_ leakage goes up with the number of testing times for gate metal defect parts, as Figure 14 shows. For parts passing HTGB 1000 h stress, repeating the I_DSS_ test merely shows no change at a fresh wafer test. Therefore, the applied compression stress after wafer processing at the gate reduces the internal electric field in AlGaN, thereby suppressing the leakage current (I_GOFF_) caused by Fowler–Nordheim (FN) tunneling when the strained device was under reverse bias [13]. However, the presence of a gate metal defect affects the controllability of the gate, leading to minimal changes in control of the peak electrical field by the gate toward the drain side. Detectability at the package-level has introduced tensile stress from packaging and electrical crowding by the gate into gate reverse bias. Therefore, the introduction of a peak electric field by the gate toward the drain site may boost the sensitivity of gate-related defect detection at the wafer-level with compressive stress.

The comprehensive fabrication qualification involved the assembly of three staggered production lots, each yielding approximately 1500 units. A thorough screening protocol was subsequently implemented at the package-level on the final devices. This package-level screening utilized a high gate-voltage stress condition, V_G_ = −60 V, specifically targeting latent defects. This initial inspection successfully identified 21 parts exhibiting the specific electrical failure signature illustrated in Figure 11b. All 21 flagged units were then subjected to the standard High-Temperature Gate Bias (HTGB) reliability test, following the established conditions depicted in Figure 6. Crucially, every single one of these screened units failed prematurely, registering failures in both I_DSS_ before the T1000 milestone, which is consistent with the failure mechanism previously observed in the smaller, preliminary HTGB sample sets. To confirm the statistical validity of the sampling plan, a comparative failure ratio analysis was performed: the sampling HTGB failure rate was 1 out of 231 (0.43%), while the package-level screening identified 21 failures out of 4269 parts (0.49%). The close correlation between these two ratios validates the fact that the samples drawn from the three qualification lots were statistically representative of the entire population’s defectivity level.

**Figure 15:**
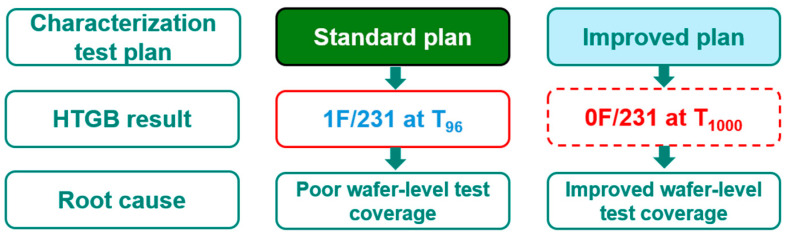
A summarized chart for the improvement of HTGB failures. The standard characterization test plan has 1 fail out of 231 parts due to poor wafer-level test coverage at the gate metal defect. In the improved characterization test plan, there are no fails out of 231 parts for 1000 h of HTGB, because the implementation of wafer-level I_DSS_ pulse tests screens out the potential gate metal defects from the foundry process.

To move beyond simple pass/fail screening and to sensitively detect subtle electrical degradation prior to packaging, a refined characterization technique was developed and deployed. This method leverages the cumulative effect of repeating I_DSS_ pulse tests to magnify the influence of minor electric field perturbations within the device structure. By plotting the I_DSS_ reading as a function of the number of test cycles, a clear pattern of I_DSS_ instability or drift could be established, as visually demonstrated in Figure 14. This enhanced characterization test plan, featuring multiple I_DSS_ pulse cycles, was subsequently implemented as a pre-packaging, wafer-level HTGB verification screen on the new production lots. After effectively filtering out and excluding the dies that exhibited I_DSS_ drifting behavior during these wafer-level pulse tests, a total of 231 non-drifting dies were randomly selected, assembled into packages, and committed to the final HTGB qualification test. The rigorous application of this new, preemptive screening methodology yielded a definitive result: there were zero failures recorded after completing the full 1000 h of HTGB stress, a significant achievement that underscores the efficacy of the improved screening protocol, as summarized in the final yield chart of Figure 15.

## 4. Conclusions

This study successfully investigated and mitigated a critical gate-related failure mechanism in power GaN HEMTs, validating the necessity of an early, sensitive wafer-level screening protocol. Failure analysis traced the HTGB T96 failure to a small-volume structural defect in the multi-layer gate metal, which was undetectable by standard SEM-EDS due to spatial resolution limitations. Comparative electrical testing revealed that the inherent high leakage current of the GaN structure renders I_GON_ insensitive to this gate defect. Conversely, I_GOFF_ showed potential, with a distinct response difference observed between the bare die and packaged device, which was successfully correlated to the package-induced tensile stress via the piezoelectric effect.

Based on these findings, we developed and implemented a multi-pulse I_DSS_ test at a high drain voltage (V_D_ = 650 V) as an effective wafer-level screening methodology. This method leverages the cumulative effect of high electrical field perturbation to amplify the subtle electrical instability caused by the gate metal defect. The rigorous application of this pre-packaging screening successfully filtered the at-risk population, resulting in zero failures out of 231 screened units after 1000 h of HTGB stress. This confirms the multi-pulse I_DSS_ test as an indispensable, high-coverage screening tool, demonstrating that early defect detection at the wafer-level significantly reduces manufacturing scrap costs and enhances the long-term reliability of GaN HEMTs for commercial applications.

## Data Availability

The datasets presented in this article are not readily available. Requests to access the datasets should be directed to Yu-Ting Chuang.
